# Activities at the Universal Protein Resource (UniProt)

**DOI:** 10.1093/nar/gku469

**Published:** 2014-06-02

**Authors:** The UniProt Consortium

The authors Rabie Saidi and Tunca Dogan were omitted from the list of the UniProt consortium in the acknowledgements section of this paper. The corrected consortium list is provided below.

## The UniProt Consortium

UniProt has been prepared by Rolf Apweiler, Alex Bateman, Maria Jesus Martin, Claire O'Donovan, Michele Magrane, Yasmin Alam–Faruque, Emanuele Alpi, Ricardo Antunes, Joanna Arganiska, Elisabet Barrera Casanova, Benoit Bely, Mark Bingley, Carlos Bonilla, Ramona Britto, Borisas Bursteinas, Wei Mun Chan, Gayatri Chavali, Elena Cibrian–Uhalte, Alan Da Silva, Maurizio De Giorgi, Tunca Dogan, Francesco Fazzini, Paul Gane, Leyla Garcia Castro, Penelope Garmiri, Emma Hatton–Ellis, Reija Hieta, Rachael Huntley, Duncan Legge, Wudong Liu, Jie Luo, Alistair MacDougall, Prudence Mutowo, Andrew Nightingale, Sandra Orchard, Klemens Pichler, Diego Poggioli, Sangya Pundir, Luis Pureza, Guoying Qi, Steven Rosanoff, Rabie Saidi, Tony Sawford, Aleksandra Shypitsyna, Edward Turner, Vladimir Volynkin, Tony Wardell, Xavier Watkins, Hermann Zellner, Matt Corbett, Mike Donnelly, Pieter van Rensburg, Mickael Goujon, Hamish McWilliam and Rodrigo Lopez at the European Bioinformatics Institute (EMBL–EBI); Ioannis Xenarios, Lydie Bougueleret, Alan Bridge, Sylvain Poux, Nicole Redaschi, Lucila Aimo, Andrea Auchincloss, Kristian Axelsen, Parit Bansal, Delphine Baratin, Pierre–Alain Binz, Marie–Claude Blatter, Brigitte Boeckmann, Jerven Bolleman, Emmanuel Boutet, Lionel Breuza, Cristina Casal–Casas, Edouard de Castro, Lorenzo Cerutti, Elisabeth Coudert, Beatrice Cuche, Mikael Doche, Dolnide Dornevil, Severine Duvaud, Anne Estreicher, Livia Famiglietti, Marc Feuermann, Elisabeth Gasteiger, Sebastien Gehant, Vivienne Gerritsen, Arnaud Gos, Nadine Gruaz–Gumowski, Ursula Hinz, Chantal Hulo, Janet James, Florence Jungo, Guillaume Keller, Vicente Lara, Philippe Lemercier, Jocelyne Lew, Damien Lieberherr, Thierry Lombardot, Xavier Martin, Patrick Masson, Anne Morgat, Teresa Neto, Salvo Paesano, Ivo Pedruzzi, Sandrine Pilbout, Monica Pozzato, Manuela Pruess, Catherine Rivoire, Bernd Roechert, Michel Schneider, Christian Sigrist, Karin Sonesson, Sylvie Staehli, Andre Stutz, Shyamala Sundaram, Michael Tognolli, Laure Verbregue and Anne–Lise Veuthey at the SIB Swiss Institute of Bioinformatics (SIB); Cathy H. Wu, Cecilia N. Arighi, Leslie Arminski, Chuming Chen, Yongxing Chen, John S. Garavelli, Hongzhan Huang, Kati Laiho, Peter McGarvey, Darren A. Natale, Baris E. Suzek, C. R. Vinayaka, Qinghua Wang, Yuqi Wang, Lai–Su Yeh, Meher Shruti Yerramalla and Jian Zhang at the Protein Information Resource (PIR).

